# Uses, misuses, new uses and fundamental limitations of magnetic resonance imaging in cognitive science

**DOI:** 10.1098/rstb.2015.0349

**Published:** 2016-10-05

**Authors:** Robert Turner

**Affiliations:** Max Planck Institute for Human Cognitive and Brain Sciences, Stephanstraße 1A, 04103 Leipzig, Germany

**Keywords:** magnetic resonance imaging, brain function, cerebral blood volume, neuroanatomy, cortical layers, myeloarchitecture

## Abstract

When blood oxygenation level-dependent (BOLD) contrast functional magnetic resonance imaging (fMRI) was discovered in the early 1990s, it provoked an explosion of interest in exploring human cognition, using brain mapping techniques based on MRI. Standards for data acquisition and analysis were rapidly put in place, in order to assist comparison of results across laboratories. Recently, MRI data acquisition capabilities have improved dramatically, inviting a rethink of strategies for relating functional brain activity at the systems level with its neuronal substrates and functional connections. This paper reviews the established capabilities of BOLD contrast fMRI, the perceived weaknesses of major methods of analysis, and current results that may provide insights into improved brain modelling. These results have inspired the use of *in vivo* myeloarchitecture for localizing brain activity, individual subject analysis without spatial smoothing and mapping of changes in cerebral blood volume instead of BOLD activation changes. The apparent fundamental limitations of all methods based on nuclear magnetic resonance are also discussed.

This article is part of the themed issue ‘Interpreting BOLD: a dialogue between cognitive and cellular neuroscience’.

## Introduction

‘Activity maps are of limited value unless they intersect with detailed neuroanatomical information’

Randlett *et al*. [1].

In the early 1990s, magnetic resonance imaging (MRI) scientists discovered that the already-known difference in magnetic susceptibility between oxygenated and deoxygenated haemoglobin could be used as an index of local brain activity [2–4]. Although the effect, whose amplitude depends both on changes in blood oxygenation and on regional cerebral blood volume (CBV), must still be considered to be somewhat empirical, considerable insight has been achieved in the past 20 years into its origins in neurovascular coupling, and its relevance to modelling of brain operations.

Because MRI is remarkably free from harmful side effects up to static field strengths of at least 8 T, the blood oxygenation level-dependent (BOLD) effect was very rapidly adopted by brain scientists who previously had access only to the somewhat invasive and spatially rather imprecise technique of positron emission tomography (PET) [5], with its unavoidable radiation dose.

In the early years of fMRI, most cognitive neuroscience studies involving imaging were performed using MRI scanners with a field strength of 1.5 T, equipped with gradient coils capable of producing gradients of only about 10 mT m^−1^, single-channel radiofrequency (RF) receiver coils and with comparatively poor temporal stability. To make the most effective use of such equipment, researchers widely adopted strategies for data analysis that in hindsight can be seen as misleading. This has become clear with the introduction of much higher field MRI scanners for human studies, up to 7 T, which have stronger magnetic field gradient coils, up to 64 channels of RF reception, and greatly improved temporal stability. The much higher signal-to-noise ratio (SNR) now makes it possible to localize brain functional activity *in vivo* within identifiable neural substrates, with reasonably well-known networks of axonal connections, allowing a game-changing approach to cognitive science and cognitive psychology.

This short review discusses the following questions:

1. What can be learned about cognition from structural and functional BOLD MRI that other techniques cannot provide?
2. What are the major flaws in current uses of fMRI?
3. Are there other ways of analysing MRI/fMRI data that provide deeper insight?
4. Are there developments in MRI and fMRI methodology that minimize the assumptions needed?
5. What are the likely fundamental limitations of all MRI methods?
6. What are the poorly explored questions relevant to fMRI?
7. What are the most synergetic other techniques?

## 1. What can be learned about cognition from structural and functional blood oxygenation level-dependent magnetic resonance imaging that other techniques cannot provide?

Many researchers take the word ‘cognition’ to mean the processes internal to the brain that culminate in the encoding of memories, planning of action or directly as immediate actions. The work involved in these processes is performed by neurons assisted by glial cells, and requires energy, which is supplied almost entirely by oxidative phosphorylation, the glucose and oxygen coming mostly from the capillaries and terminal arterioles that perfuse brain tissue [6,7]. During neuronal activity, substances are released—notably nitric oxide—that have a direct effect on local blood volume and blood flow. Although the increased metabolic demand associated with neural work results in a higher extraction of oxygen from the blood, the molecules released during synaptic activity cause expansion of the cortical arterioles and capillaries, which normally overcompensates for this increased oxygen extraction [8–10]. The end result is that the blood oxygenation typically increases, giving rise to the increase in BOLD signal observed in MRI conventionally associated with ‘brain activity’.

It is fair to claim that no other experimental technique can provide such detailed maps of human brain activity, with reasonably uniform sensitivity throughout the brain volume. The anatomical location of functional activity can be assigned by referring to structural brain images, usually so-called T1-weighted images, which can be obtained from the same subject's brain, or as an average across the brains of a group of subjects, nonlinearly warped into a suitable template brain. Reproducibility of the basic effect is well established [11], and the application of diffusion-weighting magnetic field gradients to associated MRI acquisitions allows approximate estimation of axonal connections [12]. In principle, brain structure, function and connectivity can be investigated at a spatial scale of better than 1 mm in individual human subjects. Thus, one can argue that if cognitive neuroscience is defined as the development of explanatory models of brain function based on known neuroanatomy and connectivity, BOLD fMRI is the best tool that we currently have.

However, an important question remains: what exactly do we mean by ‘brain activity’ [13]? There is now strong evidence that the amplitude of BOLD signal is well correlated with local field potential [14] and increases in gamma-band electrical activity [15], and it is quite often correlated with spike frequency [16]. However, as yet there is no unambiguous way to discriminate whether a positive BOLD signal in a given brain location arises from excitatory or inhibitory outputs from that location [17–19]. The spatial localization of increases in BOLD signal is consistent with electrocorticographic (ECoG) recordings [20] within millimetre accuracy. However, ever since the discovery of BOLD contrast it has been noted that changes in blood oxygenation owing to local changes in oxygen uptake and blood volume are carried downstream. This entails that BOLD contrast, when obtained with the most usual technique of gradient-echo MRI, is maximal at the cortical surface and in discrete pial veins [21]. Nevertheless, there is wide agreement that the BOLD signal provides fairly reliable information regarding the location of changes in brain electrical activity.

However, it is still very unclear what level of granularity needs to be considered in order to ensure the plausibility of proposed models. Over the years from 1980 until about 2010, cognitive neuroscience studies that employed neuroimaging mostly adopted a broad-brush, coarse-grain approach, inspired largely by analysis methods first developed for PET. From this perspective, typically described as statistical parametric mapping [22], the practice of spatially smoothing the raw BOLD fMRI data (acquired at approx. 3 mm resolution) to roughly the spatial resolution of processed PET data (approx. 10 mm resolution), before further analysis, was regarded as unproblematic, and indeed offered advantages in regard to sensitivity and reproducibility. At this spatial scale, useful conclusions could be drawn regarding which gyrus of the brain played a more important role in a particular brain task. Claims still continue to be made for much more precise localization within each lobe, resulting from a strategy of statistical thresholding that can deceptively produce what appear to be highly localized apparent regions of activity even when the images have been highly smoothed before analysis.

Regarding cognitive studies, strong objection [23,24] has been raised to the practice of statistical mapping of heavily smoothed and thresholded functional brain imaging data, labelling its findings as ‘neo-phrenology’ [24] and committing the mereological fallacy [23] —that is, ascribing to parts of a system attributes that can only be coherently ascribed to the entire system. A recent very well-informed critique of many aspects of current practice can be found in Shifferman [25]. It is outside the scope of this review to elaborate further on this point.

At the most general level, however, fMRI has done much to support the idea of cortical segregation, that specific brain functions can be assigned to relatively compact cortical areas that can be labelled with a description of the function. Thus, we have visual areas, auditory areas, motor and somatosensory areas and many others, which are becoming progressively subdivided as experimental designs become more subtle and imaging techniques improve. Over the past 20 years, fMRI has been able uniquely to demonstrate the fine structure of such maps [26,27], notably in elucidating the spatial structure of responses to objects at different positions in the visual field, touch and motion of different parts of the body, numerosity of visual objects [28] and variations of auditory pitch. Such studies have invariably attempted to use all the spatial resolution that fMRI can provide, avoiding spatial smoothing except for cosmetic reasons at a final stage of analysis [27]. The data obtained from such mapping experiments are likely to be important in the formulation and testing of theories of perception and motor control.

Furthermore, the concept of neuronal receptive fields [29] has recently been extended to posit the existence of population receptive fields [30], and a kindred concept, that of brain voxel encoding [31]. Such methods, reviewed by Poldrack & Farah [32], which can involve rich, quasi-naturalistic batteries of stimuli and a small number of subjects, provide us with detailed cortical maps that can be surprisingly extensive, throwing into question simplistic functional parcellations based on simpler experimental paradigms, drastic spatial smoothing and rigorous statistical thresholding. This strategy is discussed further in §3.

## 2. What are the major flaws in current uses of functional magnetic resonance imaging?

In the early days of BOLD fMRI (1990s), leading imaging neuroscience laboratories, such as the Functional Imaging Laboratory in Queen Square, London, developed the still-current methodology [33], which attempts to link brain location, neuroanatomy and function at a spatial scale of no better than 8 mm—about as close as anyone dared to expect that corresponding cortical areas could be located across brains. Standard practice included spatial smoothing of functional images by 8 mm, and group averaging. Among several strong reasons for this procedure, smoothing allowed for the residual mismatch of actual cortical areas after structural brain images had been spatially normalized into a standard template brain registered within MNI space, so that positive results could be anticipated from group averaging across normalized brains. Spatial location of activity was usually identified on a maximum probability atlas of Brodmann areas derived from the cytoarchitecture of 10 cadaver brains. Very few researchers were then aware that MRI could already be made quite sensitive to myeloarchitectural details [34].

The general linear model was used to quantify the correlation between the time course of the signal change in each voxel of the smoothed images with the applied functional paradigm. The resulting analysis packages of SPM (http://www.fil.ion.ucl.ac.uk/spm/), FSL (http://fsl.fmrib.ox.ac.uk/fsl/fslwiki/), Brain Voyager (http://www.brainvoyager.com/) and AFNI (http://afni.nimh.nih.gov/afni) continue to dominate the field of imaging-based cognitive neuroscience, with SPM still the most popular.

However, this analysis strategy entails several poorly justified assumptions [35,36], few of which are discussed in the cognitive science literature. These inevitably exclude the possibility of identifying neural competence with neuroanatomical substrate, and hence the formulation of systems neuroscience models that can benefit from prior cellular neuroscience knowledge.

In principle, the components of a system should be clearly definable, and themselves well understood. Turner [35] discusses this topic in detail, arguing that *in vivo* parcellated maps of cortex and subcortex [37], which can be acquired even at 3 T using quantitative MRI, will provide a more reliable and reproducible guide to brain components than those currently used, giving models of brain function that use our remarkably rich knowledge of neuroanatomy. A further viable addition to this may be the distinction between input and output cortical layers, probably achievable with fMRI spatial resolution of 0.5 mm or better (see §4 below).

## 3. Are there other ways of analysing magnetic resonance imaging/functional magnetic resonance imaging data that provide deeper insight?

### (a) Multivoxel pattern analysis

One way to avoid the pitfalls of premature spatial smoothing, specifically the merging together of neighbouring activations that should remain distinct because they result from different neural operations, is to use machine learning techniques to discriminate spatial patterns of brain activity specific to a particular stimulus or task from other related stimuli. This approach [38], termed multivoxel pattern analysis (MVPA), does not require spatial smoothing, and thus avoids the mistaken assumptions mentioned above. Although the ‘searchlight’ approach to MVPA of Kriegeskorte [37] effectively smooths the image data, this drawback can be avoided, as pointed out by Stelzer [39], by the use of feature weight mapping.

### (b) Voxel encoding and population receptive field mapping

One recent approach for modelling fMRI data begins with providing the experimental subject with a very large number of related stimuli or tasks, often naturalistic. These are analysed into a large set of features. The goal is to determine the functional repertoire of each grey matter voxel, as encompassed by a model that characterizes the ‘feature space’ of the stimuli. The correctness and completeness of the model in predicting brain activity to new stimuli can be tested on a separate validation dataset [31]. Such models are called encoding models, because they describe how information about the sensory stimulus is encoded in measured brain activity. Remarkable cortical maps, for instance depicting the space of semantic categories [40], have been generated using these methods. For such purposes, spatial smoothing would be quite unacceptable. Hence, this technique avoids earlier questionable assumptions, and lends itself to research in which myeloarchitecture, cytoarchitecture and functional repertoire can be directly compared.

Results using this approach reveal that specific features of experience often have widely distributed spatial representations in the brain [40]. However, clustering can also be noted, often in accordance with linguistic or common-sense categorization of experience and action. Cognitive neuroscience may benefit from deeper understanding of these data-driven insights into the categorization of experience, which may avoid the Procrustean tendency to force our experience into predefined inherited conceptual frameworks that may have little affinity with how brains actually operate [41].

The population receptive field mapping approach [30] estimates a model of the population receptive field for voxels in visual cortex that best explains measured fMRI responses resulting from a series of various visual stimuli. This can be regarded as a special case of voxel encoding, applying specifically to visual stimuli and visual cortex.

## 4. Can novel magnetic resonance imaging and functional magnetic resonance imaging methods minimize the assumptions needed?

Recent developments in MRI using the new generation of whole-body scanners at field strengths of 7 T and above have shown conclusively that submillimetre spatial resolution is now achievable for structural, functional and connectivity imaging [42–45]. For functional BOLD and structural imaging, submillimetre resolution has even been achieved with the latest generation of 3 T scanners. Such a resolution, consistent with the size of cortical columns, may constitute a critical threshold regarding realistic mechanistic explanations of brain function [46]. Furthermore, the recognition that quantitative MRI enables assessment of myelin and iron density within the living brain [47] offers a fresh outlook on systems neuroanatomy, in which a renewed study of myeloarchitecture will play a major role, and the interaction between brain iron, dopamine and neuromelanin can be explored in the context of brain function [48].

### (a) Brain structure

Use of high-field MRI in human brain, particularly at 7 T, enables *in vivo* individual-specific maps of genuine cortical microstructure [49,50], which can be correlated with cortical function in the same brain [51,52]. Quantitative structural maps of the longitudinal relaxation time T1 [52] of entire brains can be obtained with better than 0.5 mm isotropic resolution, which closely resemble myelin-stained histological sections at low-resolution [35,53]. High-quality structural data revealing myelin content can also be achieved at 3 T [54,55], but with correspondingly lower spatial resolution. Cécile & Oskar Vogt [56], pioneers in myeloarchitecture research in the first half of the twentieth century, showed that there is good concordance between structural parcellations of the cortex based on myeloarchitecture and on cytoarchitecture [57,58].

Hence, ‘*in vivo* Brodmann mapping’ [35,59,60] can be performed using MRI-observable differences in grey matter myelination. Cortical areas known from post-mortem studies to be heavily myelinated such as primary motor, somatosensory, auditory, visual cortex [61] and area V5-MT [62] are easily discriminated from surrounding less-myelinated regions. Moreover, surface registration [63] across subjects of T1 maps of the cortex can be achieved efficiently and precisely, impressively matching corresponding cortical areas. Using the high spatial resolution available at 7 T, and a realistic algorithm modelling the effect of cortical folding on layer position [64], this matching provides cortical profiles of myelination comparable to the Vogt histological findings [56]. The higher spatial resolution available using prospective motion correction [65], together with precise averaging of individual subject brains across multiple imaging sessions, will enable still finer discriminations of cortical areas. MRI techniques that can measure dendritic density [66] and capillary density will also assist in this endeavour.

De Martino [45] has recently shown that functional and structural data for auditory cortex can be elegantly combined, to suggest that primary auditory cortex can be pragmatically defined as the region of high myelination (short T1) on the crown of Heschl's gyrus in the temporal lobe. Thus, like can now be compared with like in group studies—averaging of structural and functional results can be performed in an area-wise manner, without spatial smoothing. This matching of function and structure also offers the potential to integrate the findings of systems neuroscience with those of cellular neuroscience, for instance to explain the functional competence of a cortical area in terms of its neuronal makeup and configuration.

The rebirth of scientific interest in myeloarchitecture [67] holds the promise of deeper insights into principles of cortical organization. Once the location of changes in brain activity in a given subject's brain can be identified via their own native myelin-based cortical atlas, the corresponding cytoarchitecture can be looked up in a concordance atlas. When combined with high-quality crossing-fibre dMRI tractography such information could greatly assist mechanistic explanation of brain function. With the achievable isotropic resolution of 300 µm in structural MR images, there are no more than a few thousand pyramidal neurons within each voxel. In brain locations showing columnar structure, many of such neurons are likely to participate in network activity cooperatively.

The high spatial resolution of long echo-time gradient-echo structural phase images at 7 T has an additional benefit. In the form of neuromelanin in the basal ganglia, iron provides excellent contrast-to-noise ratio, and hence their precise delineation. Quantitative maps of magnetic susceptibility give even clearer pictures [68–70] of these under-researched structures, vital to human life.

### (b) Brain function

As mentioned above, work at 7 T in recent years has produced remarkable improvements in functional imaging (reviewed by Van der Zwaag [44]), to the level of 0.5 mm isotropic resolution [42,45,71]. Use of T2-weighted three-dimensional gradient-recalled echo and spin-echo imaging has become an option for very high spatial resolution [71], owing to its high SNR per unit time and relative insensitivity to larger draining veins. Parallel acquisition, both in-plane [42] and simultaneous multislice [72,73] has been the mainstay of 7 T fMRI, allowing images of high spatial resolution and good image quality to be obtained with remarkable speed. Functional studies are proceeding even with small deep structures such as the subthalamic nucleus [74].

However, perhaps the most exciting development in fMRI at high field is the implementation of high-sensitivity methods for measuring changes in regional CBV. These methods [75] use a preparatory inversion pulse to null the MRI signal from the blood, leaving a signal from brain tissue alone which varies linearly with the blood volume, and thus with the state of brain activity. A modification of this technique enables submillimetre resolution at 7 T [76]. CBV appears to be locally controlled by pericytes responsive to activity in neighbouring neurons within the thickness of the cortex [77–79]. Thus, the ability to monitor CBV non-invasively in real time may greatly improve investigation of variations of neural activity at the resolution of the cortical layer [80–83]. Further evidence for the layer-specificity of CBV mapping comes from a study [84] of the olfactory bulb in rat brain, which shows good separation of CBV response for stimuli which differentially excite neurons in specific cortical layers.

### (c) Brain connectivity

Brain connectivity can be approximately evaluated using diffusion-weighted imaging [85,86] and by analysing spatial correlations in task-absent BOLD signal [87] (so-called resting state). Diffusion-weighted imaging uses large magnetic field gradient pulses between spin excitation and data acquisition to label water molecular motions [88]. Work at 7 T with gradients of 80 mT m^−1^ [43,89] gives excellent delineation of fibre orientations, with spatial resolution up to 0.8 mm isotropic. Here, fibre tracts can be seen to bend into the sulcal banks, as they should, in contrast with the more standard spatial resolution of 3 mm, where computed tracts appear to terminate on the gyral crowns. However, diffusion imaging has important limitations in depicting brain connections (see the critical papers of Jones [86] and Thomas *et al.* [90]).

Functional connectivity studies at 7 T, which benefit greatly from the improved SNR and resolution, are increasing in number. For an excellent primer, see Power [91], and for current examples, see Raemakers [92]. There is still considerable controversy regarding optimal methods for extracting connectivity information [93] and the neural processes underlying the observable resting state networks remain somewhat obscure [94]. However, their patterns are considered to be useful heuristic guides to brain connectivity, even to the point of providing an alternative method for cortical parcellation [95].

### (d) Layer-dependent functional magnetic resonance imaging

**Figure 1.**
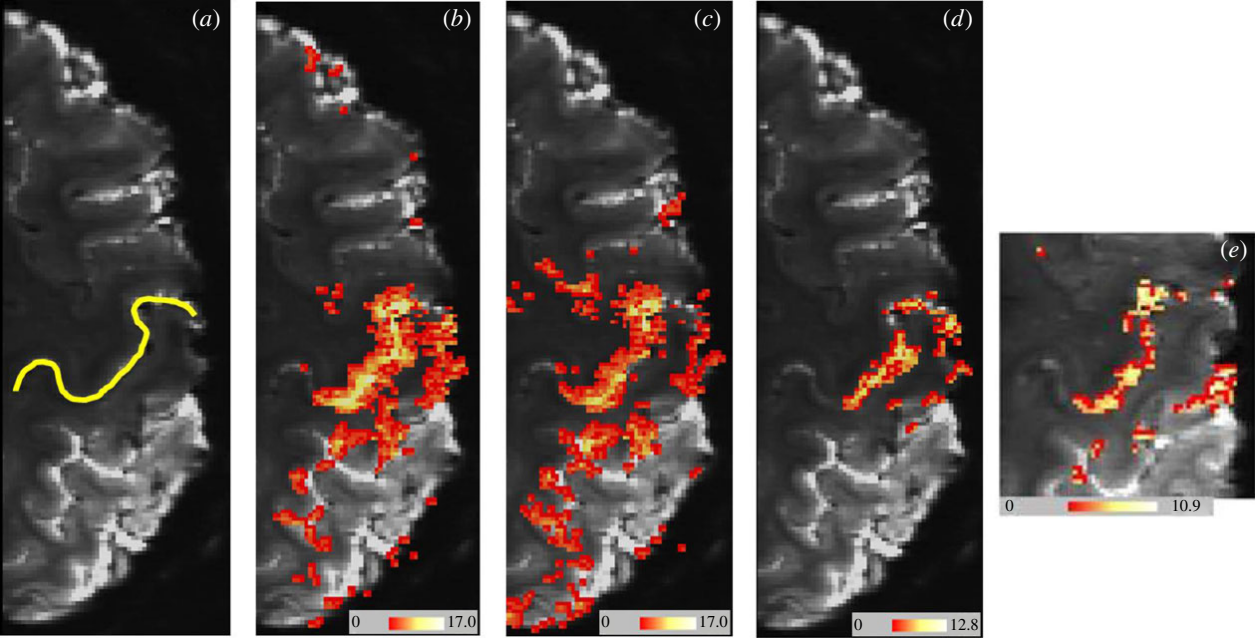
Single subject axial section images acquired with echo planar imaging slices, with colour-overlaid BOLD activation maps during finger movement and imagined finger movement (figure courtesy of Robert Trampel). Spatial resolution 0.75 mm isotropic. (*a*) Axial section acquired using zoomed EPI shows raw data quality. The yellow line indicates the central sulcus, with ‘hand knob’. (*b*) Activation map during ‘tapping’ versus ‘rest’. (*c*) ‘Moving’ versus ‘rest’. (*d*) Contrast of ‘tapping’ versus ‘moving’. (*e*) Activation map during imagined finger movement. Colour bars indicate *z*-scores. Functional maps thresholded at *p* < 0.05, using false discovery rate.

**Figure 2.**
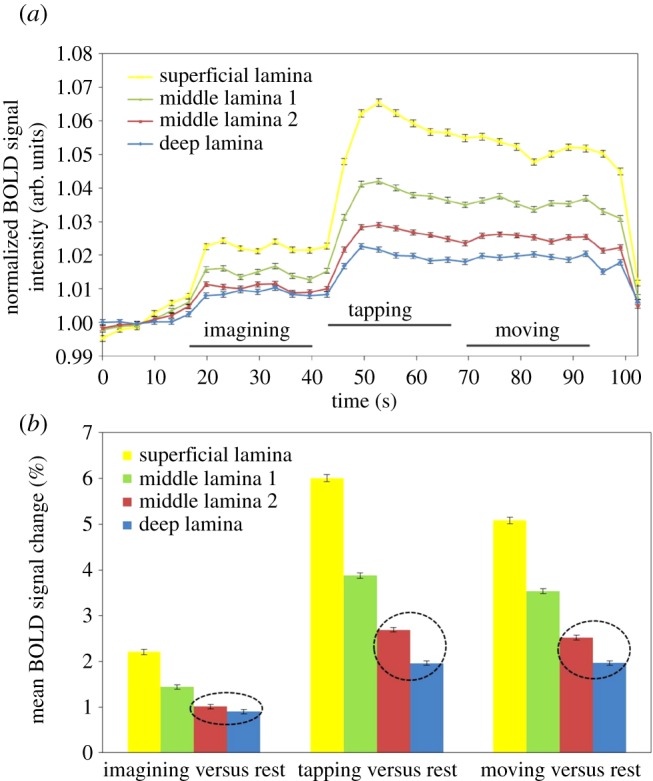
Layer-dependence of BOLD activation for finger tapping, finger movement and imagined finger tapping. Grand average time course and mean BOLD signal of nine subjects at four different cortical depths in primary motor cortex. (figure courtesy of Robert Trampel). (*a*) Grand average BOLD time courses obtained at four cortical depths in primary motor cortex averaged across nine subjects. Error bars represent standard error of the mean. (*b*) Corresponding mean BOLD signal difference between the three conditions (‘imagining’, ‘tapping’, ‘moving’) and ‘rest’, respectively. Error bars represent standard error of the mean. The dashed circle emphasizes the smaller difference between ‘middle lamina 2’ and ‘deep lamina’ for motor imagery compared to actual motor performance.

The submillimetre spatial resolution available at 7 T for functional imaging of human brain allows investigation of variations of BOLD contrast across the thickness of the cortex. In a pioneering study in 2011, Trampel *et al.* [96] measured activation in the hand area of human primary motor cortex. They used gradient-echo BOLD fMRI to study activation for three motor tasks: finger tapping, finger movement without touch and motor imagery. The primary motor cortex was unambiguously identified by its anatomical location and high myelin content, as indicated by its characteristically short T1. At 7 T, structural data were obtained with 0.5 mm isotropic resolution, and fMRI data with 0.75 mm isotropic resolution (figures 1 and 2). Cortical activation profiles specific to each motor condition were computed, and averaged across the activated area at four different cortical depths, and across nine human volunteer subjects. During the motor imagery condition, lacking motor output from layer V of the primary motor cortex, the BOLD signal at a depth corresponding to this cortical layer was found to be reduced (figure 2), by comparison with the signal from other cortical layers in this condition.

As mentioned in §1, however, the BOLD signal represents the history of blood oxygenation changes as blood travels from the pial arteries into the diving arterioles and thence into capillaries and veins. As such, this signal cannot provide a precise layer-specific indication of oxygen extraction. In the study just described, the statistically highly significant difference found in cortical profiles between the tapping and motor imagery conditions is noteworthy, but should not be over-interpreted. (See further discussion in §5a(ii) below.)

## 5. What are the likely fundamental limitations of all magnetic resonance imaging methods?

Most MRI acquisitions depend on the interaction of the minute magnetic moments of the protons comprising the nuclei of hydrogen atoms in water molecules with feasible applied magnetic fields—static, audiofrequency and radiofrequency. It is only the vast abundance of these protons in brain tissue that enables any NMR signal to be observable.

Increasing the static field incurs increasingly severe problems of RF engineering, RF safety, perceptible physiological effects, subject acceptability, high gradient strength requirements and expense. At 7 T, where several of these problems now have adequate solutions, the best structural whole-brain spatial resolution achievable in a scanning session of 1 h is likely to be about 300 µm isotropic. Higher resolution may be achieved with part-brain acquisition, with averaging across sessions to achieve adequate SNRs. Prospective motion correction [65,97] will be required to ensure image quality.

In regard to functional studies, the magnetic fields associated with coherent neural activity, while easily observable using magnetoencephalography techniques, are too small to be localized using MRI methods [98]. So the only currently practical way that MRI can contribute to studies of brain function is via its sensitivity for the vascular response to neural activity.

The MR signal associated with this vascular response can depend on the blood's velocity, volume fraction and oxygenation. MRI sequences can be designed to be sensitive to one or more of these parameters, but there are fundamental limitations.

### (a) Specific limitations of blood oxygenation level-dependent

#### (i) Ambiguity

The BOLD signal is a non-quantitative index of changes in both blood volume and oxygen extraction. A quantitative measure of changes in oxygen utilization, or brain work, would be more desirable. While this can be estimated by combining BOLD measurements with cerebral blood flow measurements, following Davis [99], the weak link is the poor sensitivity of CBF measurement by MRI. The sensitivity is improved at 7 T, but at this field strength another fully quantitative measure of brain activity becomes feasible, non-invasive measurement of CBV using a modification of vascular space occupancy (VASO) [82,100].

#### (ii) Poor layer-specificity

Blood volume is apparently controlled by resistance arterioles and pericytes, with little functional change in pial veins, and is thus spatially quite well matched to demand, but changes in blood oxygenation and blood flow are more non-local [21]. Gradient-echo (GE) BOLD changes are maximal at the cortical surface, and may even be detected in pial veins several millimetres downstream from the active grey matter. To minimize this problem, some researchers advocate spin-echo BOLD, but its sensitivity is much lower than GE-BOLD, even at 7 T [101,102]; and even with spin-echo acquisition, much BOLD signal arises from principal intracortical veins [103,104]. Because most of the signal arises from larger venules and surface veins, the effective GE-BOLD resolution in the plane of the cortex cannot be better than the spacing of principal intracortical veins (about 0.7 mm) [10,105,106]. In addition, the cortical profile of BOLD signal represents a spatial convolution of task-driven changes in oxygen extraction with local blood flow, modulated by changes in blood volume, which blurs out the layer-dependence of underlying neural activity. Capillary perfusion, as measured using arterial spin labelling (ASL), should, in principle, be well localized to neural activity [107], but the relatively low sensitivity of this technique in humans has deterred its widespread usage.

A recent simplified model of the blurring effect [108] predicts a cortical depth effect that roughly fits experimental data, confirming that the cortical profile of the BOLD signal cannot be naively interpreted as a profile of neuronal activity. An fMRI study concerning layer-specific feedback in visual cortex [109] suggests that where sufficient spatial separation exists between top-down and bottom-up input layers, BOLD contrast may still be enough to discriminate their characteristic patterns of activity.

## 6. What are the poorly explored questions relevant to functional magnetic resonance imaging?

### (a) Direction of causation

Graph or network models of brain function can only make testable predictions if they include a measurable variable describing the direction of causation between separate cortical or subcortical areas. This is accessible neuroanatomically only in cadaver brain, using anterograde and retrograde tracer methods.

The graph theory-based dynamic causal modelling approach attempted to simplify the causality problem by invoking neural mass modelling, but lacks neuroanatomical realism [110]. Sadly, the experimental variance explained by the best-fitting graph rarely exceeds only a few per cent [110]. Hence, the results have very little predictive power, and consequently little scientific value.

As an alternative approach to the causality question, some researchers [96,111] advocate the use of prior neuroanatomical knowledge of neuronal circuitry. Histology and animal brain research can define the specific cortical layers in which input and output pathways terminate. In principle, activity in input layers can be driven by experimental conditions, and the behavioural effects of activity in output layers can be experimentally observed. Where input and output cortical layers are distinguishable by fMRI, causal relationships between brain areas could thus be empirically validated. Similarly, where the input layers of top-down and bottom-up afferents are spatially separated, a causal direction could be established for the neural activity corresponding to a given task. The Trampel *et al.* study [96] summarized above (figures 1 and 2) relies on the fact that the output to the corticospinal tract of motor nerves from agranular primary motor cortex M1 arises almost entirely from large pyramidal neurons in layer V. Thus, in the motor imagery condition, with no motor output, one might expect a comparatively lower activation signal from layer V—as was indeed observed. Fortunately, the cortical thickness in M1 is unusually large, about 4 mm, which facilitated the discrimination of specific cortical layers using fMRI.

Other experimental paradigms offer themselves for this type of study. For instance, primary auditory cortex is well known to be activated by auditory input, but also strongly modulated by auditory imagery [112]. Sensory input to auditory cortex arrives in layer IV, whereas top-down modulation involves neurons in layers I and VI [112]. Similar mismatches of input and output layers are found in other primary sensory areas. Research with human subjects is particularly valuable in this context, because of our remarkable compliance and proficiency with the type of laboratory tasks required to tease out differential activity across the cortical thickness.

**Figure 3.**
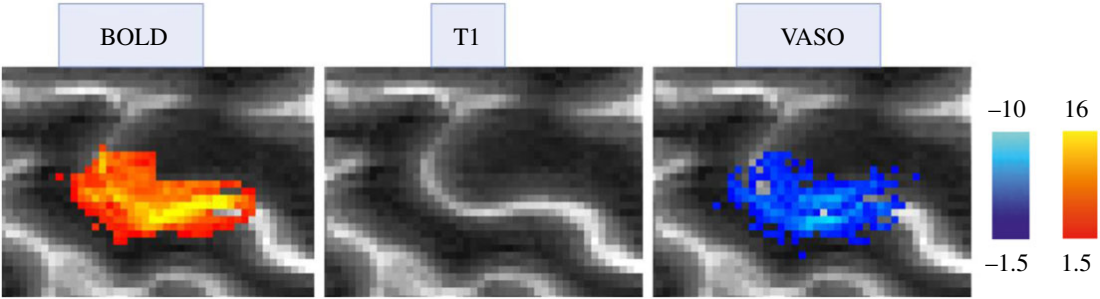
Functional MRI using BOLD contrast and cerebral blood volume maps acquired with slab-selective vascular occupancy imaging (VASO). Comparison of single-subject activation maps generated by finger tapping, in an axial section through the central sulcus. Echo-planar data acquisition, resolution 0.74 × 0.74 × 2 mm^3^, TE = 20 ms, TR = 1.5 s (interleaved VASO and BOLD acquisition; figure courtesy of Laurentius Huber).

However, as noted previously, BOLD fMRI can provide at best a blurry, smeared-out account of layer-dependent activity, because of the cross-layer ‘bleed’ of oxygenation changes. The recent work of Huber [82], enabling enhanced sensitivity of CBV measurements at high field, offers an alternative form of functional imaging in which the variation of the signal with cortical depth more closely matches the expected neuronal activity (figure 3), without the maximum at the cortical surface found with BOLD imaging. As commented in §4(b), this is consistent with local control of blood volume, as suggested by the intracortical distribution of pericytes [79].

### (b) Prevalence of columnar organization

Moon *et al.* [105] compared the spatial specificity of BOLD and CBV measurements in studies of columnar structure in feline cortex, showing clear benefits for CBV. A recent fMRI study of cortical columns in auditory cortex [113] further encourages a more detailed exploration of the granularity of the human cortex, which may vary with brain area and function. Is columnar organization a universal principle [114,115]? Or does experience-driven cortical self-organization result in columnar organization only in specific areas in which this wiring strategy is optimal? In order to focus efforts using MRI to investigate such questions, a deeper understanding of cortical functional anatomy, based on animal studies and post-mortem human brain histology, would be helpful.

### (c) Sparse encoding versus population encoding

ECoG studies in entorhinal cortex [116] show that individual neurons, each responsive to a wide range of visually presented examples of a single object or person, are sparsely distributed within this cortical area. Even if a BOLD signal from such an area could be obtained from presentation of a particular object, this does not mean that the entire area represents that object, or even class of objects. By contrast, spatially mapped cortical areas such as retinotopic areas conversely use population encoding as an important computational principle. Research with invasive techniques on animal models will surely reveal many more instances of each type of encoding—which ultimately might be found to correlate with the distinctive cyto- and myeloarchitecture of specific brain regions.

## 7. What are the most synergetic other techniques?

Currently, in the view of the author, the most exciting non-invasive technique for quantitative studies of cognition in human brain is the measurement of CBV, using MRI blood-nulling techniques. With the adequate SNR available at high magnetic field, this offers the hope of layer-specific identification of induced neural activity. Careful comparisons with spin-echo BOLD and three-dimensional gradient and spin-echo BOLD are urgently needed, together with combined electrocorticographic and VASO animal brain studies with multicontact electrodes.

Advanced histological studies may also shed light on the optimal functional MRI technique for exploring layer-dependent activity. The key question is: is there a correlation between the distributions of pericytes, which locally control blood flow and hence blood volume, and mitochondria, which generate the ATP molecules essential for the various neurochemical and neuroelectrical processes that constitute functional activity?

Cadaver brain cortical sections can be selectively stained to reveal these components. Mitochondrial density can be inferred quite well using stains for cytochrome oxidase, and pericytes can be selectively stained [79]. Good spatial correlation would suggest that cortical profiles of changes in CBV map the layer dependence of neuronal function. Here, ‘neuronal activity’ would be compactly defined as activity that costs energy (see discussion in §5a(i) above). Early work by Borowsky [117] showed good qualitative correlation between capillary density and cytochrome oxidase staining in several regions of rat brain. This suggests the need for much more comprehensive studies in cadaver human brain, in which cortical profiles of pericyte density, cytochrome oxidase density and capillary density are all compared.

## 8. Summary and conclusions

This review has described some of the historical development of MRI-based neuroimaging techniques currently used in cognitive science research. Weaknesses in the most popular analysis strategies are identified. Game-changing developments in MRI and fMRI capabilities are then discussed that show great promise for bridging the gap between cellular and systems neuroscience. At magnetic field strengths of 7 T and above, data with submillimetre resolution can be acquired in scan times consistent with using human subjects. To depict the human cerebral cortex in such detail with MRI can be considered a threshold that allows cortical areas to be structurally discriminated, layer-dependence of functional activity to be determined, and much-improved characterization of axonal pathways to be estimated. Novel methods for analysis are tracking these technical improvements, enabling new penetrating questions regarding the organization of brain function.

Layer-specific fMRI offers promise in defining the directions of causation between brain areas in specific tasks. This aim requires the use of prior histological knowledge of neural circuitry in each cortical area involved, and carefully defined hypotheses and experimental protocols. The optimal acquisition technique for layer-specific fMRI is not yet fully established. Histological studies comparing pericyte and mitochondrial distributions should offer insight regarding the value of high-resolution mapping of CBV changes.

Ultimately, because feasible fMRI techniques all depend on the neurovascular response, still deeper understanding will be needed of the geometry of the cortical and subcortical microvasculature, the molecular signals relating neural electrical activity to vasodilation and vasoconstriction, the spatial distribution of pericytes, the details of oxygen extraction and the integration of the control mechanisms of the cerebral circulation.
